# Wayfinding in Interior Environments: An Integrative Review

**DOI:** 10.3389/fpsyg.2020.549628

**Published:** 2020-11-06

**Authors:** Saman Jamshidi, Mahnaz Ensafi, Debajyoti Pati

**Affiliations:** ^1^Department of Design, College of Human Sciences, Texas Tech University, Lubbock, TX, United States; ^2^Department of Building Construction, College of Architecture and Urban Studies, Virginia Polytechnic Institute and State University, Blacksburg, VA, United States

**Keywords:** wayfinding, indoor environment, spatial cognition, spatial behavior, integrative review

## Abstract

Wayfinding is an issue in complex facilities—including hospitals, airports, and office buildings—and wayfinding difficulties are associated with negative psychological and physiological consequences. In addition, since finding one’s way in a building is a prerequisite for successfully using that building, wayfinding has attracted the attention of scholars and decision makers. The goal of this article is to review and synthesize the published literature on wayfinding in interior environments. A systematic search was conducted of four databases: PsychINFO, JSTOR, ProQuest, and EBSCO. A hand search was also conducted. From the initial harvest of 804 records, a total of 84 records met the inclusion criteria for full review. After several rounds of review, four broad domains were identified: (1) wayfinding cognition, (2) wayfinding behavior, (3) individual and group differences, and (4) environmental factors. These domains are used as a framework to organize the findings, and the review shows that the sub-domains most thoroughly addressed in the literature are spatial memories, floor plan configuration, landmarks, signs, and maps. This review can deepen the field’s understanding of factors that contribute to interior wayfinding and can serve as a resource for decision makers and designers.

## Introduction

Being able to find one’s way in a building is a prerequisite for successfully fulfilling one’s goal in that building (Weisman, 1981). The process of determining a route from one location to another and navigating that route is referred to as “wayfinding” (Chen et al., 2009). Wayfinding and navigation have been used interchangeably in the literature (e.g., McNamara et al., 2008). However, Montello (2005) proposed that navigation consists of wayfinding and locomotion. He distinguished between wayfinding, as the decision-making process of determining a route to a destination, and locomotion, as the act of actually moving on a route (Montello, 2005). Some researchers also used navigation and locomotion interchangeably (e.g., Karimi, 2015).

In this paper, wayfinding is defined as a problem-solving process (Arthur and Passini, 1992) of determining and navigating a route to a destination and recognizing the destination as approaching it (Chen et al., 2009). Accordingly, wayfinding is an outcome of both cognitive functions (such as problem-solving and decision making) and behaviors (such as navigation and decision execution) (Arthur and Passini, 1992). Wayfinding can be challenging for many people, especially in complex buildings like hospitals, airports, and office buildings (Arthur and Passini, 1992). It can be especially challenging in stressful situations (e.g., Schmitz, 1997; Kallai et al., 2007), such as when one is ill or under time constraints. Because stress is known to impair information processing (Broadbent, 1971), it can be assumed that stressful situations impair the cognitive aspect of wayfinding (e.g., Schmitz, 1997; Thomas et al., 2010). Wayfinding can also be especially difficult for individuals with physical or mental limitations, including individuals with vision impairments, limited physical mobility, or reduced cognitive functioning.

Wayfinding problems have been associated with negative physical and psychological effects (Carpman and Grant, 2002). For example, Shumaker and Reizenstein (1982) pointed out that in healthcare settings, wayfinding problems can lead to confusion, frustration, anger, stress, elevated blood pressure, headaches, and fatigue. Wayfinding problems have also been found to negatively affect how people view businesses (e.g., shopping centers and hospitals) and to cause visitors to interrupt staff for help finding their way (Arthur and Passini, 1992).

While much of the variance in individuals’ wayfinding performance can be accounted for by factors intrinsic to people (e.g., their spatial abilities) (e.g., Kirasic, 2000), a growing body of literature shows that environmental factors—such as the complexity of a building’s layout (e.g., Slone et al., 2015), landmarks (e.g., Davis et al., 2008), and the attributes of corridors (e.g., Vilar et al., 2014b, 2015) also play critical roles in the wayfinding process. While a substantial body of literature exists pertaining to distinct and separate phenomena associated with wayfinding, no study has collated the available empirical evidence into a single source of knowledge, which prompted the current literature review. This review offering a foundation for future studies and design decisions. For a review of wayfinding theories in interior environments, refer to Jamshidi and Pati (2020).

## Method

To identify relevant articles, a systematic search was conducted in the PsychINFO, JSTOR, ProQuest, and EBSCO online databases. The search terms were “wayfinding” and “way finding.” Using the same keywords, a hand search was also conducted of journals related to the subject which were the Journal of Environmental Psychology, Environment and Behavior, and the journal of Spatial Cognition and Computation. The assistance of an expert in cognitive neuroscience was used to include additional key studies related to wayfinding cognition in the hand search process.

No specific timeframe was established for the search process. Once the initial list of records had been generated, a three-stage review process was used to identify studies that met each of the inclusion criteria. The three stages of this process were (1) a title review, (2) an abstract review, and (3) a full-text review, and studies were included if and only if they (1) were focused wholly or in part on wayfinding, (2) were conducted in interior environments, (3) were empirical and (4) written in English. Studies were excluded if their subjects were children, if their subjects were individuals who were visually impaired or physically or mentally disabled, and if they focused on technology testing.

**FIGURE 1:**
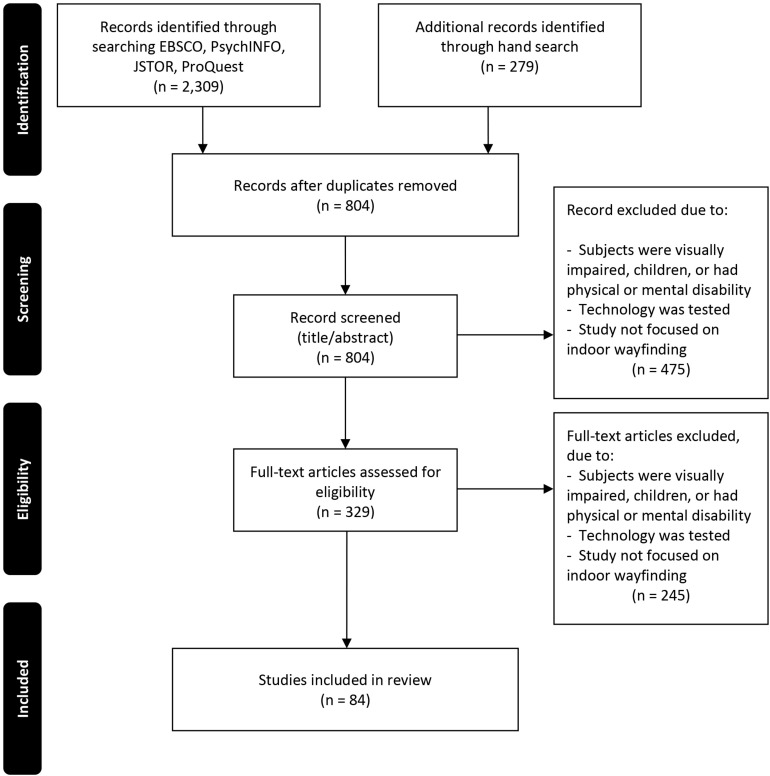
PRISMA search strategy flow diagram. From Moher et al. (2009).

The initial list of records included 2,309 articles obtained via the database search and 279 articles obtained via the hand-search. After duplicates were removed, 804 articles were included in the three-stage review process. Eighty-four studies published between 1982 and 2019 satisfied all of the inclusion criteria, and the findings of these studies are reported in this review. A graphical representation of the search strategy is presented in Figure 1.

Although several classifications for wayfinding exist (e.g., Wiener et al., 2009; Carlson et al., 2010; Dalton et al., 2019), they focus on just some aspects of wayfinding. Thus, a new framework was needed to organize findings in this paper. Included studies were reviewed to identify the broad factors that each study examined. In an iterative classification process, several domains and sub-domains pertaining to the broad factors emerged. The idea was not to create a theoretical classification of conceptual structures of all the articles based on concepts in wayfinding and cognition, but to create a framework where different intersection of domains of knowledge can be attached. Using this framework, studies were clustered based on domains of knowledge and investigation. Studies were organized in the narrative by reporting their findings regarding two sub-domains (hereafter called intersecting domain) at a time. A single study might have examined more than two sub-domains. In this case, results regarding each pair of sub-domains were reported separately under appropriate subheadings.

A three-person team of environmental design researchers (the authors) who are architects or interior designers, conducted the search, review, and synthesis between November 2017 and December 2018. During this process, any disagreements among the authors were resolved through discussion. All team members had prior experience and training in conducting literature reviews.

## Findings

**FIGURE 2:**
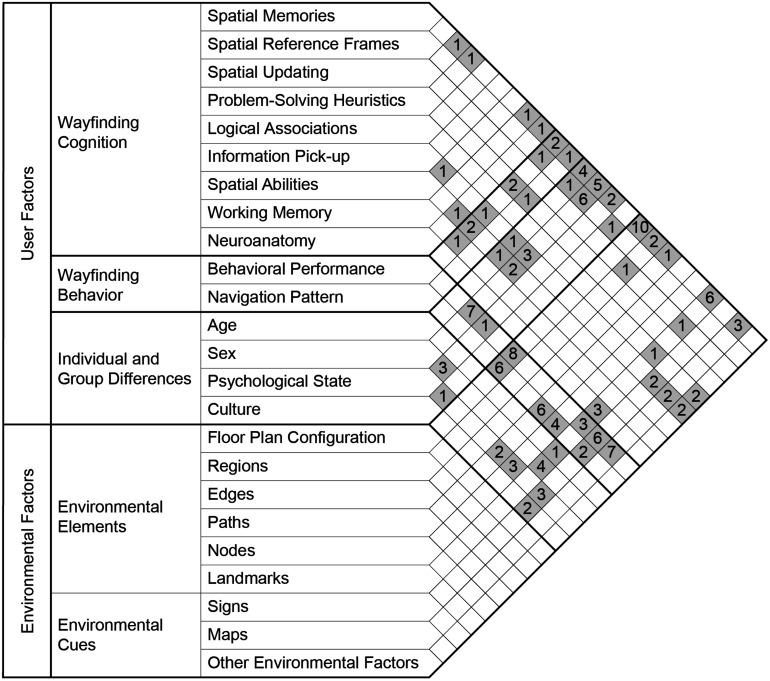
Relationships between different sub-domains (i.e., intersecting domains) are shown in this folded matrix. The numbers in the cells indicate the number of articles in each intersecting domain. Some studies examined more than two sub-domains; hence they can fit into multiple pairs of sub-domains.

Two broad factors were identified in the reviewed studies which are (a) user factors and (b) environmental factors. User factors were classified into three domains which are (1) wayfinding cognition, (2) wayfinding behavior, (3) individual and group differences. Environmental factors have two domains which are (1) environmental elements and (2) environmental cues. Any of these domains have sub-domains which can be seen in Figure 2. Definitions of sub-domains are provided in Table 1. The following review reports findings of the 84 studies included in the review. It begins by reporting the results of studies into the relationships between sub-domains of different user factors, starting with wayfinding cognition followed by wayfinding behavior, and individual and group differences. Then, it presents the findings of studies that specifically examined the role of environmental factors in the user factors (i.e., wayfinding cognition, wayfinding behavior, and individual and group differences). The list of actual studies in each intersecting domain was shown in Table 2.

**TABLE 1:** Two broad factors were identified in the reviewed studies: (a) user factors and (b) environmental factors. These factors were classified into further domains and sub-domains. Definitions of sub-domains and the rationale of the classification is provided in this table.

Major Factors	Domains	Sub-domains	Definitions	Descriptions
User Factors	Wayfinding Cognition	Spatial Memories	Spatial memories “are memories of the locations of objects, places, and environmental features” (McNamara, 2013, p. 174).	“Cognitive function is a broad term that refers to mental processes involved in the acquisition of knowledge, manipulation of information, and
		Spatial Reference Frames	A spatial reference frame is a system for representing the spatial relationships between entities in space (Klatzky, 1998)	reasoning. Cognitive functions include the domains of perception, memory, learning, attention, decision making, and language abilities” (Kiely, 2014,
		Spatial Updating	Spatial updating refers to the continuous computation of self-to-object relationships performed by a navigator (Zhong and Kozhevnikov, 2016)	p. 975). These sub-domains were clustered under wayfinding cognition domain based on the rationale that they are mental processes and relates to one or more of the aforementioned general cognitive
		Spatial Problem-Solving Heuristics	Spatial problem-solving heuristics are mental shortcuts that reduce an individual’s cognitive load. They require a minimum of information and allow individuals to make decisions in short periods of time (Bobadilla-Suarez and Love, 2018).	functions. For example, spatial memories and working memories relate to memory. Spatial reference frames relate to spatial memory and hence memory. Spatial updating relates to reasoning and manipulation of information. Spatial
		Logical Associations	Logical associations refer to people’s inferences regarding typical “links between object types and certain functions or regions in buildings” (Frankenstein et al., 2012).	problem-solving heuristics relate to decision making. Logical associations relate to reasoning. Information pick-up relate to perception and attention. Spatial abilities relate to general cognitive
		Information Pick-Up	Information pick-up refers to the act of selecting spatial information from alternatives offered by the environment (Neisser, 1976).	ability. Neuroanatomy is not a cognitive function. However, it was categorized under wayfinding cognition based on the rationale that neuroanatomy
		Spatial Abilities	“Spatial abilities are tied to performance on spatial aptitude tests and the dimensions of visualization and orientation contained within those tests” (Golledge and Stimson, 1997, p. 156).	pertains to the domain of knowledge in which the neural correlation with cognitive functions are investigated.
		Working Memory	“Working memory is the ability that allows us to retain limited amounts of information for a short amount of time while we are actively working on that information” (Banich and Compton, 2011, p. 293).	
		Neuroanatomy	“Neuroanatomy is the description of the parts of the nervous system encompassing the brain, spinal cord, peripheral nervous system and nerves” (Lyle, 2011, p. 1011).	
	Wayfinding Behavior	Behavioral Performance	An individual’s behavioral performance is a measure of how well people perform in navigating the environment during wayfinding tasks as measured by time, traveled distance, and speed. This is the authors’ definitions in this paper.	These two behavioral sub-domains are relevant to wayfinding only if they are demonstrated during wayfinding. Base on this rationale, behavioral performance and navigation pattern were clustered under wayfinding behavior.
		Navigation Pattern	Navigation pattern refers to the general preference of people to choose a certain physical space or zone during movement in an environment or at decision points. This is the authors’ definitions in this paper.	
	Individual/Group Differences	Age	Reviewed papers looked at young versus old people.	These sub-domains were clustered under individual and groups differences based on the rationale that
		Sex	Reviewed papers looked at males versus females.	they look at wayfinding differences between groups
		Psychological State	The psychological state refers to the stress, anxiety, and spatial anxiety that people might experience in regular or emergency situations.	of people (e.g., males versus females) or individuals (e.g., people with higher versus lower levels of spatial anxiety)
		Culture	Reviewed papers looked at the differences between people of different nations.	
Environmental	Environmental	Floor Plan Configuration	The general layout of a building (Weisman, 1981).	Regardless of the actual physical forms,
Factors	Elements	Regions	Regions are sections of a building that has some common characteristics, and people recognize regions within a building and mentally move into them. This concept was adapted from Lynch’s (1960) definition of districts in outdoor environments.	environments consist of fundamental elements that can be achieved through various physical forms. These elements were clustered under environmental elements domain. These elements can have different attributes. For example, configuration of a floor plan (as an environmental
		Edges	Adapted from Lynch’s (1960) definition of edges in outdoor environments, in this paper edges are defined as boundaries between two regions in interior environments that are not considered paths.	element) can be complex (i.e., attribute) or a landmark can be salient.
		Paths	Adapted from Lynch’s (1960) definition of paths in outdoor environments, in this paper paths are defined as channels in interior environments that people typically or potentially move along.	
		Nodes	A node is a location in which two or more alternatives are available (O’Neill, 1991a).	
		Landmarks	Landmarks are objects, places, and environmental attributes that serve as reference points (Lynch, 1960).	
	Environmental Cues	Signs	Signs are elements that provide directional information in an environment (O’Neill, 1991a).	Environmental cues are among environmental factors that signal users during wayfinding to find or recognize a target.
		Maps	“Diagrammatic, 2-dimensional representation of the global environment” (Pati et al., 2015, p. 50).	
		Other Environmental Factors	“Elements or attributes of physical environment procured or designed by interior designers used in a different way by subjects” (Pati et al., 2015, p. 50).	

**TABLE 2:** The list of actual studies in each intersecting domain.

Section Title	Intersecting Domains	Qty.	Citation
Spatial memories.	Spatial memories	3	Levine et al., 1982; Montello and Pick, 1993; Anooshian, 1996
The relationship between spatial memories – and spatial updating.	Spatial memories – spatial updating	1	Zhong and Kozhevnikov, 2016
The relationship between spatial memories – and working memories.	Spatial memories – working memories	1	Labate et al., 2014
The relationship between spatial memories – and neuroanatomy	Spatial memories – neuroanatomy	1	Bohbot et al., 2004
The relationship between spatial memories – and behavioral performance	Spatial memories – behavioral performance	2	Hölscher et al., 2006; Padgitt and Hund, 2012
The relationship between spatial memories and navigation pattern	Spatial memories – navigation pattern	1	Kallai et al., 2007
The relationship between spatial memories – and age.	Spatial memories – age	4	Kirasic, 1991; Wilkniss et al., 1997; Kirasic, 2000; Davis et al., 2008
The relationship between Sspatial memories – and sex	Spatial memories – sex	5	Schmitz, 1997, 1999; Castelli et al., 2008; Choi et al., 2006; Cherney et al., 2008
The relationship between spatial memories – and psychological state	Spatial memories – psychological state	2	Schmitz, 1997; Thomas et al., 2010
The relationship between spatial reference frames and behavioral performance	Spatial reference frame – behavioral performance	1	Münzer and Stahl, 2011
The relationship between spatial reference frames – and age	Spatial reference frame – age	1	Rodgers et al., 2012
The relationship between spatial reference frames – and sex	Spatial reference frame – sex	6	Lawton, 1994, 1996; Lawton et al., 1996; Lawton and Kallai, 2002; Chen et al., 2009; Rodgers et al., 2012
The relationship between spatial reference frames – and culture	Spatial reference frame – culture	1	Lawton and Kallai, 2002
The relationship between spatial updating – and spatial memories	Spatial updating – spatial memories	1	Zhong and Kozhevnikov, 2016
The relationship between problem-solving heuristics – and spatial memories	Problem-solving heuristics – spatial memories	1	Hölscher et al., 2006
The relationship between problem-solving heuristics – and behavioral wayfinding performance	Problem-solving heuristics – behavioral performance	2	Hölscher et al., 2006, 2009
The relationship between problem-solving heuristics – and navigation pattern.	Problem-solving heuristics – navigation pattern	1	Van Tilburg and Igou, 2014
The relationship between information pick-up – and spatial ability	Information pick-up – spatial ability	1	Padgitt and Hund, 2012
The relationship between information pick-up – and behavioral performance	Information pick-up – behavioral performance	1	Padgitt and Hund, 2012
The relationship between information pick-up – and age.	Information pick-up – age	1	Lee and Kline, 2011
The relationship between information pick-up – and sex.	Information pick-up – sex	3	Chebat et al., 2005; Picucci et al., 2011; Rosenthal et al., 2012
The relationship between spatial ability – and information pick-up	Spatial ability – information pick-up	1	Padgitt and Hund, 2012
The relationship between spatial ability – and neuroanatomy	Spatial ability – neuroanatomy	1	Wegman et al., 2014
The relationship between spatial ability – and behavioral performance	Spatial ability – behavioral performance	2	Hund and Nazarczuk, 2009; Padgitt and Hund, 2012
The relationship between spatial ability – and age.	Spatial ability – age	1	Kirasic, 2000
The relationship between spatial ability – and sex.	Spatial ability – sex	2	Lawton, 1996; Picucci et al., 2011
The relationship between working memory – and spatial memories	Working memory – spatial memories	1	Labate et al., 2014
The relationship between working memory – and behavioral performance	Working memory – behavioral performance	1	Hund, 2016
The relationship between behavioral performance – and working memory	Behavioral performance – working memory	1	Hund, 2016
The relationship between behavioral performance – and sex	Behavioral performance – sex	7	Schmitz, 1997; Moffat et al., 1998; Choi et al., 2006; Chen et al., 2009; Hund and Padgitt, 2010; Kober and Neuper, 2011; Rosenthal et al., 2012
The relationship between behavioral performance – and psychological state.	Behavioral performance – psychological state	1	Schmitz, 1997
The relationship between psychological state – and sex.	Psychological state – sex	3	Schmitz, 1997, 1999; Lawton and Kallai, 2002
The relationship between psychological state – and culture.	Psychological state – culture	1	Lawton and Kallai, 2002
The relationship between floor plan configuration – and spatial memories	Floor plan configuration – spatial memories	10	O’Neill, 1991c, 1992; Haq and Zimring, 2003; Baskaya et al., 2004; Werner and Schindler, 2004; Jansen-Osmann et al., 2007; Abu-Obeid and Abu-Safieh, 2010; Slone et al., 2015; Li and Klippel, 2016; Lu and Ye, 2019
The relationship between floor plan configuration – and behavioral performance	Floor plan configuration – behavioral performance	8	O’Neill, 1991b, c, 1992; Werner and Schindler, 2004; Jansen-Osmann et al., 2007; Hölscher et al., 2012; Slone et al., 2015; Li and Klippel, 2016
The relationship between floor plan configuration – and navigation pattern	Floor plan configuration – navigation pattern	6	Peponis et al., 1990; Haq, 2003; Haq and Zimring, 2003; Hölscher et al., 2006, 2012; Lu and Ye, 2019
The relationship between regions– and spatial memories	Regions– spatial memories	2	Wang and Brockmole, 2003; Montello and Pick, 1993
The relationship between regions– and spatial updating	Regions– spatial updating	1	Mou and Wang, 2015
The relationship between edges – and spatial memories.	Edges – spatial memories	1	Buckley et al., 2016
The relationship between paths – and navigation pattern	Paths – navigation pattern	6	Butler et al., 1993; Frankenstein et al., 2012; Hidayetoglu et al., 2012; Wiener et al., 2012; Vilar et al., 2014b, 2015
The relationship between paths – and psychological state	Paths – psychological state	2	Vilar et al., 2013, 2014b
The relationship between nodes – and navigation pattern	Nodes – navigation pattern	4	Vilar et al., 2012, 2014b, 2015; Van Tilburg and Igou, 2014
The relationship between nodes – and psychological state	Nodes – psychological state	3	Tang et al., 2009; Vilar et al., 2013, 2014b
The relationship between landmarks – and spatial memories	Landmarks – spatial memories	6	Jansen-Osmann and Wiedenbauer, 2004; Jansen-Osmann and Fuchs, 2006; Davis et al., 2008; Hidayetoglu et al., 2012; Werkhoven et al., 2014; Sameer and Bhushan, 2015
The relationship between landmarks – and spatial updating	Landmarks – spatial updating	1	Wan et al., 2012
The relationship between landmarks – and logical associations.	Landmarks – logical associations	1	Frankenstein et al., 2012
The relationship between landmarks – and neuroanatomy.	Landmarks – neuroanatomy	3	Janzen and Jansen, 2010; Kober and Neuper, 2011; Sharma et al., 2017
The relationship between landmarks – and behavioral performance	Landmarks – behavioral performance	3	Jansen-Osmann and Wiedenbauer, 2004; Werkhoven et al., 2014; Sharma et al., 2017
The relationship between landmarks – and age.	Landmarks – age	1	Davis et al., 2008
The relationship between landmarks – and sex.	Landmarks – sex	4	Schmitz, 1997, 1999; Choi et al., 2006; Kober and Neuper, 2011
The relationship between sign – and information pick-up	Sign – information pick-up	2	Rousek and Hallbeck, 2011; Hashim et al., 2014
The relationship between sign – and behavioral performance	Sign – behavioral performance	6	O’Neill, 1991b; Butler et al., 1993; Wright et al., 1993; Cope et al., 1999; Chen et al., 2009; Vilar et al., 2014a
The relationship between sign – and navigation pattern.	Sign – navigation pattern	2	Vilar et al., 2014b, 2015
The relationship between sign – and psychological state	Sign – psychological state	3	Tang et al., 2009; Vilar et al., 2014b, 2015
The relationship between sign – and culture	Sign – culture	2	Hashim et al., 2014; Joy Lo et al., 2016
The relationship between maps – and spatial memories	Maps – spatial memories	3	Thorndyke and Hayes-Roth, 1982; Golledge et al., 1995; Richardson et al., 1999
The relationship between maps – and information pick-up	Maps – information pick-up	2	Meilinger et al., 2006; Hölscher et al., 2009
The relationship between maps – and behavioral performance.	Maps – behavioral performance	7	Levine et al., 1984; Butler et al., 1993; Wright et al., 1993; Meilinger et al., 2006; Chen et al., 2009; Hölscher et al., 2009; Münzer and Stahl, 2011
The relationship between other environmental factors – and logical associations.	Other environmental factors – logical associations	2	Frankenstein et al., 2012; Pati et al., 2015
The relationship between other environmental factors – and information pick-up	Other environmental factors – information pick-up	2	Pati et al., 2015; Ghamari and Pati, 2018

### Wayfinding Cognition

Nine major sub-domains of wayfinding cognition were identified in the literature: (1) spatial memories, (2) spatial reference frames, (3) spatial updating, (4) spatial problem-solving heuristics, (5) logical associations, (6) information pick-up, (7) spatial ability, (8) working memory, and (9) neuroanatomy (see Table 1 for definitions). This review focuses on wayfinding cognition within interior environments, hence studies regarding general spatial cognition, that were not conducted in interior environments, were not included. For a review of general spatial cognition, refer to Waller and Nadel (2013).

#### Spatial Memories

Most of the studies that examined spatial memories adopted the classification provided by Siegel and White (1975). According to Siegel and White (1975), spatial memories include three levels of spatial knowledge: landmark knowledge, route knowledge, and survey knowledge. Landmark knowledge is knowledge of objects and places based on their appearances or subjective importance in the environment, “without knowing their relative spatial relationship” (Iachini et al., 2009, p. 228; McNamara, 2013). Route knowledge is sequence knowledge connecting objects or places (Siegel and White, 1975). Survey knowledge (or configurational knowledge) is “knowledge of the overall spatial layout of the environment” and of the spatial relationships between objects and places (Cubukcu and Nasar, 2005; McNamara, 2013, p. 175).

Anooshian (1996) compared participants’ acquired spatial knowledge across two learning conditions: (a) a place condition, in which participants were asked to learn landmarks while moving down a path, and (b) a turn condition, in which participants were asked to learn the correct turns while landmarks were present. The results revealed that the participants in the place condition generated better landmark sequence knowledge (i.e., recalling landmarks in the order that they were exposed with) and survey knowledge than did the participants in the turn condition. Further analysis also showed that spatial memories seem to be disconnected from each other and can be described as independent pieces of spatial information. Montello and Pick (1993) suggested that individuals can integrate survey knowledge acquired from two routes learned separately. They found that when subjects were given a verbal description that revealed the spatial relationships between two floors of a building that they had learned separately, they were able to point to landmarks on the other floor—an indicator of the acquisition of survey knowledge—albeit with less accuracy than when they pointed to landmarks on the same floor. An important concept related to spatial memories are Cognitive maps, that are “spatial representations that contain qualitative metric information about large-scale environments, and which can be used to generate novel shortcuts or to take detours” (Weisberg and Newcombe, 2016, p. 768). In a series of experiments, Levine et al. (1982) found that cognitive maps acquired by participants had two characteristic of visual images: (1) “simultaneous representation of sequentially placed points and (2) orientation” (p. 157). They therefore concluded that cognitive maps are picture-like. Sixteen studies were found that examined the relationships between spatial memories and spatial updating, working memories, neuroanatomy, behavioral performance, navigation pattern, age, sex, and psychological state.

##### The relationship between spatial memories and spatial updating

Evidence suggests that there is a link between the formation of survey knowledge and the use of path integration (Zhong and Kozhevnikov, 2016). Path integration, as one form of spatial updating, refers to determining the location of an invisible target by estimating the traveled distance and direction (Mou and Wang, 2015). In a study by Zhong and Kozhevnikov (2016), participants’ use of path integration and their acquisition of survey knowledge were measured (via self-report and sketch mapping, respectively) after they had completed route-learning tasks in several buildings. The sketch maps revealed that the participants had acquired two distinct types of survey knowledge: (a) egocentric survey knowledge (i.e., maps representing survey knowledge and aligned with the initially learned path) and (b) allocentric survey knowledge (i.e., maps representing survey knowledge and not aligned with the initially learned path). The participants who demonstrated egocentric survey knowledge also reported greater use of path integration during route learning.

##### The relationship between spatial memories and working memories

Labate et al. (2014) used a dual-task paradigm to examine the engagement of two types of working memories in the acquisition of survey knowledge: verbal working memories and spatial working memories. The results revealed that while both types of working memories were involved in most of the measures of survey knowledge (e.g., a pointing task between floors and a map-completion task), spatial working memory had a greater implication in the acquisition of survey knowledge (e.g., pointing task within floor).

##### The relationship between spatial memories and neuroanatomy

Neuroscience studies suggest that different memory systems are responsible for handling two types of spatial memories: (1) survey knowledge (knowledge of the relationships between landmarks) and (2) route knowledge (which is a form of sequential knowledge acquired based on rewarded responses to stimuli) (Bohbot et al., 2004). Bohbot et al. (2004) found that the hippocampal system plays a critical role in survey knowledge (cognitive map) and that the caudate nucleus is associated with route knowledge.

##### The relationship between spatial memories and behavioral performance

Hölscher et al. (2006) found that participants who were familiar with a building (i.e., had spatial memories of it) were able to navigate to a target in the building using the shortest and fastest route. However, for people who are not familiar with a building, providing different levels of spatial knowledge may yield different behavioral outcomes. For example, Padgitt and Hund (2012) found that participants who used route descriptions (left/right descriptions and landmarks) made fewer behavioral errors in wayfinding tasks than did participants who used survey descriptions (descriptions using cardinal directions and distances).

##### The relationship between spatial memories and navigation pattern

Neophobia (anxiety caused by new environments) may affect individuals’ navigation behavior during wayfinding (Kallai et al., 2007). Kallai et al. (2007) observed the navigation patterns of people in a circular arena and found that they preferred to stay close to the border of a maze and avoided navigating the inner area in the early and middle phases of learning a new place. This pattern disappeared as the participants grew familiar with the environment.

##### The relationship between spatial memories and age

Aging is associated with declines in several cognitive functions (e.g., Deary et al., 2009; Salthouse, 2009). The literature suggests that aging may also affect spatial cognitive functions. For example, Davis et al. (2008) used a virtual environment (VE) to compare the performance of younger adults (aged 18–35) and older adults (aged 65+) in place learning, (i.e., “the cognitive process involved in encoding the cognitive map,” Davis et al., 2008, p. 253). The results revealed that the older adults demonstrated greater heading error—i.e., the angular deviation of one’s heading from one’s target—suggesting that the older adults demonstrated less effective place leaning than did the younger adults. Although this study was conducted in VE, it has implications in interior wayfinding. These results are supported by the results of Kirasic (2000), which suggest that older adults acquire less spatial knowledge (i.e., knowledge of landmarks, routes, distances, and directions). Studies also suggest that older adults are slower than younger adults in acquiring spatial knowledge (Kirasic, 1991) and in performing spatial tasks (Davis et al., 2008). However, Wilkniss et al. (1997) found that although older adults performed worse than their younger counterparts in memorizing routes and struggled to remember the temporospatial order of landmarks, the two groups demonstrated equal ability to recognize landmarks.

##### The relationship between spatial memories and sex

Males and females appear to differ in their ability to acquire different levels of spatial memories. Although Castelli et al. (2008) found no sex differences in participants’ performance on a route-learning test (an indicator of route-knowledge acquisition), they found that the male participants made fewer errors than did the female participants in a pointing task (an indicator of survey-knowledge acquisition), suggesting that the male participants acquired greater survey knowledge than did the female participants. Castelli et al. (2008) also found that performance on a landmark-positioning test (which used a map of a previously experienced VE) and performance on a pointing test (an indicator of survey knowledge) were significantly correlated only for males, suggesting that the male and female participants may have used different strategies in the landmark positioning test (Castelli et al., 2008).

Choi et al. (2006) examined sex differences in the correlation between wayfinding strategy (a landmark-biased strategy vs. a cardinal-biased strategy) and wayfinding performance (measured via behavioral measures like distance, frequency of hesitations, and errors) by asking participants to give direction descriptions of a route they had experienced. The results revealed that the female participants were more likely than the male participants to use a landmark-biased strategy and that the use of this strategy was associated with better wayfinding performance only for the female participants (no such relationship was observed for the male participants). These results suggest that the female participants acquired spatial memories more effectively when they used a landmark-biased strategy. Other studies found similar results (Schmitz, 1997, 1999; Cherney et al., 2008). In two studies by Schmitz (1997, 1999), the female participants referred more frequently to landmarks than did the male participants in drawing maps and giving directions, while the male participants used both landmarks and route directions (e.g., turn left/right).

##### The relationship between spatial memories and psychological state

Schmitz (1997) found that anxiety affects spatial memory: participants who reported higher levels of anxiety recalled more landmarks than route directions (e.g., turns at decision points) when they were asked to draw a map and write directions through a maze (although this result was not statistically significant). Thomas et al. (2010) found that stress has different effects in men and women on the neurological systems responsible for cognitive-map guided and landmark-guided navigation. Cognitive-map guided navigation depends on spatiotemporal encoding and correlates with hippocampal activation; and landmark-guided navigation relates to route following and correlates with caudate nucleus activation (Thomas et al., 2010). They found that in the female participants, stress disrupted only the cognitive system responsible for cognitive-map-guided navigation and had no effect on landmark-guided navigation. They also found that stress had no effect on the cognitive-map and landmark-guided navigations of the male participants (Thomas et al., 2010).

#### Spatial Reference Frames

A spatial reference frame is a system for representing the spatial relations of entities in space (Klatzky, 1998). There are two types of spatial reference frames: (1) egocentric frames relate to the axes of an individual’s body, and (2) allocentric reference frames, which are independent of the individual’s body and perspective (O’Keefe and Nadel, 1978). Seven studies were found that investigated the relationship between spatial reference frames and behavioral performance, age, sex, and culture.

##### The relationship between spatial reference frames and behavioral performance

Münzer and Stahl (2011) examined the impact on wayfinding performance of three learning conditions: (1) an allocentric visualization that shows a route on maps, (2) an egocentric visualization that consists of a series of images of decision points, and (3) an egocentric visualization that consists of a virtual walk of the route. They found no difference between the behavioral performance of participants in allocentric and egocentric visualizations. However, participants performed better in the virtual walk condition compared to the condition in which they were exposed to a series of images of decision points.

##### The relationship between spatial reference frames and age

Rodgers et al. (2012) used a virtual Y-maze task to determine the reference-frame preferences of older and younger adults. They found that while younger adults use both types of reference frames in equal proportion, older adults tend to rely more heavily on egocentric reference frames (Rodgers et al., 2012). Although this study was conducted in VE, it has implications in interior wayfinding.

##### The relationship between spatial reference frames and sex

Rodgers et al. (2012) found no sex difference in spatial-reference-frame preference, but several other studies found that women tend to prefer egocentric reference frames and men tend to prefer allocentric reference frames (Lawton, 1994, 1996; Lawton et al., 1996; Lawton and Kallai, 2002; Chen et al., 2009).

##### The relationship between spatial reference frames and culture

In a cross-cultural study including American and Hungarian participants, Lawton and Kallai (2002) found that male participants were more likely to use global reference frames such as cardinal reference frames (global reference frames are one kind of allocentric reference frame) and female participants were more likely to use route strategies (route strategies often relies on the egocentric reference frame).

#### Spatial Updating

“Spatial updating” refers to a navigator’s continuous computation of self-to-object relationships (Zhong and Kozhevnikov, 2016). Spatial updating incorporates two strategies: (1) piloting (i.e., using visible spatial entities to determine the location of an invisible target) and (2) path integration (i.e., determining the location of an invisible target by estimating the traveled distance and direction) (Mou and Wang, 2015). One study was found that examined the relationship between spatial updating and spatial memories.

##### The relationship between spatial updating and spatial memories

Evidence suggests an association between the formation of survey knowledge and the use of path integration (Zhong and Kozhevnikov, 2016). For further details, refer to the “The relationship between spatial memories – and spatial updating” section.

#### Problem-Solving Heuristics

Heuristics are mental shortcuts that reduce an individual’s cognitive load by enabling them to use a minimum of information to make quick decisions (Bobadilla-Suarez and Love, 2018). However, because heuristics ignore parts of the environmental information (Bobadilla-Suarez and Love, 2018), they can produce errors. Studies have identified seven general heuristics used by individuals to make wayfinding decisions: (1) the action continuation heuristic, (2) the initial segment heuristic, (3) the least-decision-load heuristic, (4) the least-angle heuristic, (5) the central point heuristic, (6) the hill-climbing heuristic, and (7) the fine-to-coarse heuristic.

The action continuation heuristic states that when none of one’s alternatives seem beneficial or necessary, one proceeds with one’s current course of action (Van Tilburg and Igou, 2014). The initial segment heuristic states that one chooses the initial path that enables one to postpone changing one’s path for as long as possible (Van Tilburg and Igou, 2014). The least-decision-load heuristic states that one chooses a series of “paths with the least number of possible decision points” (Spiers and Maguire, 2008, p. 233). The least-angle heuristic states that one chooses paths that proceed toward one’s goal with a minimum of angular deviation (Hochmair and Frank, 2000). The central point heuristic states that one uses well-known parts of a building that are considered as the skeleton of a building (e.g., main corridors and entry halls) (Hölscher et al., 2006). The hill-climbing heuristic states that one chooses actions that yield immediate progress toward one’s target by accomplishing easily obtainable subgoals (Braisby and Gellatly, 2012; Van Tilburg and Igou, 2014). Finally, the fine-to-coarse planning heuristic states that one divides one’s environment into different regions, plan coarsely when navigating between regions, and plan in fine details when navigating within a given region (Spiers and Maguire, 2008).

Particularly in multilevel buildings, special cases of the least-angle heuristic and the fine-to-course planning heuristic were described in the literature: the direction heuristic and the floor heuristic. According to Hölscher et al. (2006), the direction heuristic (a special case of the least-angle heuristic) states that one first moves toward one’s target, then change one’s level. In contrast, the floor heuristic (a special case of the fine-to-course planning heuristic), states that one first moves to the target’s floor, then move toward the target (Hölscher et al., 2006). Three studies were found that examined the relationships between problem-solving heuristics and spatial memories, behavioral performance, and navigation patterns.

##### The relationship between problem-solving heuristics and spatial memories

Hölscher et al. (2006) analyzed the use of three heuristics (the floor, direction, and central-point heuristics) among participants who were either familiar with or unfamiliar with a multilevel building and found that while the participants who were familiar with the building were more likely to use the floor heuristic, the participants who were unfamiliar with the building were more likely to use the central-point heuristic.

##### The relationship between problem-solving heuristics and behavioral performance

Hölscher et al. (2006) compared the use of three heuristics in a multi-level building (the floor, direction, and central point heuristics) and found that the participants who used the floor heuristic demonstrated the best wayfinding performance (i.e., chose the shortest path and reached the target in the shortest amount of time), while the participants who used the direction heuristic demonstrated the worst wayfinding performance. However, these results are contradicted by the results of Hölscher et al. (2009), who found that participants who first moved to the correct part of the building and then moved to the appropriate level demonstrated better wayfinding performance than did participants who used the floor heuristic. To resolve the conflict between the results of these two studies, the authors explained that the studies produced different results because they were conducted in buildings with different properties. While the first study (Hölscher et al., 2006) was conducted in a multi-level building, the second study (Hölscher et al., 2009) was conducted in multiple multi-level buildings. The researchers concluded that in both studies, hierarchical planning was the most effective, so which strategy is best depends on the spatial properties of the building (Hölscher et al., 2009).

##### The relationship between problem-solving heuristics and navigation pattern

In a series of studies, Van Tilburg and Igou (2014) examined four heuristics for participants’ preference—the action-continuation heuristic, the initial segment heuristic, the least-angle heuristic, and the hill-climbing heuristic—in navigating a maze with equally functional routes at a choice point. They found that the participants’ behavior displayed the action-continuation heuristic.

#### Information Pick-Up

“Information pick-up” refers to the act of selecting spatial information from among alternatives offered by the environment (Neisser, 1976). Five studies were found that investigated the relationship between information pick-up and spatial ability, behavioral performance, age, and sex.

##### The relationship between information pick-up and spatial ability

Padgitt and Hund (2012) examined the relationship between individuals’ self-reported sense of direction and the level of knowledge they preferred to receive in direction descriptions, and they found that while participants who self-reported better sense of direction preferred direction descriptions that provided survey knowledge (e.g., knowledge of cardinal directions and distances), people who reported lesser sense of direction preferred direction descriptions that provided route knowledge (e.g., left–right descriptors and landmarks).

##### The relationship between information pick-up and behavioral performance

Studies show that when people are provided certain types of information (e.g., verbal directions), the level of knowledge this information provides can affect their wayfinding behavioral performance. For example, Padgitt and Hund (2012) found that participants who were provided with route descriptions (left–right descriptors and landmarks) made fewer behavioral errors in performing wayfinding tasks than did participants who were provided with survey descriptions (cardinal directions and distances).

##### The relationship between information pick-up and age

Studies suggest that older adults tend to pick up different environmental information than do younger adults. Lee and Kline (2011) used Virtual Reality (VR) to examine the impact of two architectural wayfinding aids on the wayfinding performance of older and younger adults and found that the older participants tended to pick up environmental information with a higher level of saliency (e.g., a big logo and a wall with different colors) than did the younger participants. Although this study was conducted in VE, it has implications in interior wayfinding.

##### The relationship between information pick-up and sex

Studies suggest that men and women tend to use different sources of information during wayfinding. Chebat et al. (2005) examined wayfinding in a shopping mall and found that the male participants used more landmarks while the female participants were more likely to ask other people. Two other studies examined sex preferences toward the use of two types of environmental cues: geometry cues and landmark cues. In these studies, geometry cues were understood as cues related to the general shape of the environment. Rosenthal et al. (2012) found that the wayfinding performance of female participants improved when they were provided with landmark cues and that the male participants performed better when they were provided with geometry cues. Picucci et al. (2011) found that although the female participants preferred to use landmark cues rather than geometry cues, they performed as well as the male participants when provided with only geometry cues. They also found that the male participants were able to integrate both types of environmental cues (Picucci et al., 2011).

#### Spatial Ability

“Spatial abilities are tied to performance on spatial aptitude tests and the dimensions of visualization and orientation contained within those tests” (Golledge and Stimson, 1997, p. 156). Among the most common spatial abilities measured in the literature were sense of direction and mental rotation. Six studies were found that examined the relationship between spatial ability and information pick-up, neuroanatomy, behavioral performance, age, and sex.

##### The relationship between spatial ability and information pick-up

Padgitt and Hund (2012) found participants who reported strong sense of direction preferred route descriptions that provided survey knowledge, while participants who reported weak sense of direction preferred route descriptions that conveyed route knowledge.

##### The relationship between spatial ability and neuroanatomy

Wegman et al. (2014) examined the relationship between self-reported navigation ability and neuroanatomy and found that good navigators had greater volumes of gray matter in the right anterior parahippocampal gyrus/rhinal cortex than did bad navigators, and that bad navigators had greater volume in the right caudate nucleus.

##### The relationship between spatial ability and behavioral performance

A number of studies have explored the relationship between sense of direction and wayfinding behavioral performance, and they found that participants with strong sense of direction were faster and committed fewer errors than did participants with weaker sense of direction (Hund and Nazarczuk, 2009; Padgitt and Hund, 2012).

##### The relationship between spatial ability and age

Kirasic (2000) found that age-related differences in environmental learning were mediated by general spatial abilities. In Kirasic’s (2000) model, environmental learning predicts wayfinding behavior (Kirasic, 2000). Since spatial abilities (such as mental rotation and visualization) decline with age (e.g., Hertzog and Rypma, 1991; Dobson et al., 1995), according to this model, declines in general spatial abilities (as measured by psychometric tests) partially explain the reduced wayfinding performance of older adults.

##### The relationship between spatial ability and sex

Studies have found that women report less confidence in wayfinding than men do (Lawton, 1996; Picucci et al., 2011).

#### Working Memory

“Working memory is the ability that allows us to retain limited amounts of information for a short amount of time while we are actively working on that information” (Banich and Compton, 2011, p. 293). Two studies were found that examined the role of working memory in spatial memories and behavioral performance.

##### The relationship between working memory and spatial memories

While both verbal and spatial working memory were found to be involved in the acquisition of survey knowledge, spatial working memory had greater implications (Labate et al., 2014) (for additional details, refer to the “The relationship between spatial memories – and working memories” section).

##### The relationship between working memory and behavioral performance

Hund (2016) used a dual-task paradigm to examine the effects of verbal and visuospatial working memory on wayfinding performance and found that performing a visuospatial task during wayfinding resulted in an increase in time needed for participants to perform the wayfinding task suggesting that visuospatial working memory is more important in fast wayfinding.

#### Neuroanatomy

“Neuroanatomy is the description of the parts of the nervous system encompassing the brain, spinal cord, peripheral nervous system and nerves” (Lyle, 2011, p. 1011). Two studies were found that investigated the relationships between neuroanatomy and spatial memories, spatial reference frames, and spatial abilities. Regarding spatial memories, Bohbot et al. (2004) showed that the hippocampal system plays a critical role in survey knowledge (cognitive map) and that the caudate nucleus is associated with route knowledge. Regarding spatial ability, Wegman et al. (2014) found that participants who self-reported higher levels of navigation ability tended to have greater volumes of gray matter in the right anterior parahippocampal gyrus/rhinal cortex, while participants who self-reported lower levels of navigation ability had greater volume in the right caudate nucleus.

### Wayfinding Behavior

This section reports the findings of studies into the navigation as the behavioral aspect of wayfinding. Two aspects of wayfinding behavior were identified: (a) behavioral performance (i.e., how well people perform in navigating the environment during wayfinding tasks as measured by time, traveled distance, and speed), and (b) navigation pattern (i.e., the general preference of people to choose a certain physical space or zone during movement in an environment or at decision points.).

#### Behavioral Performance

The following section reports the findings of eight studies that examined the relationships between behavioral performance and working memory, sex, and psychological state.

##### The relationship between behavioral performance and working memory

While both verbal and visuospatial working memory have been found to contribute to wayfinding, visuospatial working memory plays a stronger role (Hund, 2016) (for additional details, refer to the “The relationship between working memory – and behavioral performance” section).

##### The relationship between behavioral performance and sex

Several studies found no sex differences in wayfinding behavioral performance (e.g., Hund and Padgitt, 2010), but these results do not show that men and women use the same cognitive processes to complete wayfinding tasks. While Kober and Neuper (2011) found no differences between men and women on measures of behavioral wayfinding performance in navigating a VE (e.g., traveled distance and recognized targets), EEG data revealed an increase in the theta frequency bands of the female participants compared to the male participants. These results suggest that although men and women may perform similarly on behavioral measures, their underlying cognitive processes may differ.

Other studies found behavioral differences between men and women. Schmitz (1997) found that men finished a maze task faster than women did. Similarly, Moffat et al. (1998) found that men solved a virtual maze faster than women did (although this study was conducted in VE, it has implications in interior wayfinding), and Choi et al. (2006) found that when instructed to return to the origin of a task via the shortest possible route, men took shorter routes than women did.

Among the other factors that influence wayfinding performance is the stereotype that men are better than women at wayfinding (Rosenthal et al., 2012). Rosenthal et al. (2012) found that male participants who were informed of the sex-difference stereotype performed better than did male participants in a control group, but being informed of the stereotype had no effect on female participants. In addition, evidence suggests that sex differences can be eliminated. For example, Chen et al. (2009) examined the effect of you-are-here (YAH) maps and signage on the wayfinding performance of men and women and found that although the men outperformed the women overall, the sex difference disappeared when signage was provided as a wayfinding support system.

##### The relationship between behavioral performance and psychological state

Anxiety can worsen behavioral performance. Schmitz (1997) found that women experienced more anxiety than men did and that participants with higher anxiety completed a maze task more slowly.

### Individual and Group Differences

Four sub-domains were identified pertaining to individual and group differences: age, sex, culture, and psychological state. The findings regarding age, sex, and culture are discussed in detail in the previous sections. The following section reviews the findings regarding psychological state.

#### Psychological State

Studies show that spatial anxiety can affect other wayfinding-related functions such as spatial memories (Schmitz, 1997), navigation strategy (Thomas et al., 2010), and behavioral performance (e.g., speed and hesitation) (Schmitz, 1997). In addition, neophobia can affect navigation behavior (Kallai et al., 2007). Studies have examined the relationships between psychological states and spatial memories, sex, and culture, but most of the findings of these studies were reported in the previous sections. The following section reports only findings on the relationship between psychological states and sex and culture

##### The relationship between psychological state and sex

Studies suggest that women experience more spatial anxiety than men do (Schmitz, 1997, 1999; Lawton and Kallai, 2002). For example, Schmitz (1997) found that female participants reported greater anxiety than did male participants and that participants (regardless of their sex) who experienced greater anxiety recalled more landmarks than route directions (e.g., turns at decision points) when asked to create a map of and written directions through a maze (though this result was not significant). In light of these results, the author concluded that anxiety may partially explain sex differences in wayfinding.

##### The relationship between psychological state and culture

In a cross-cultural study of participants in the United States and Hungary, Lawton and Kallai (2002) found that women experienced greater spatial anxiety than did men and that American women reported greater anxiety than did Hungarian women.

### Environmental Factors

Nine environmental factors were identified in the literature: regions, edges, paths, nodes, landmarks, floor plan configuration, signs, maps, and other environmental factors. For the purpose of this paper, the first five factors were adapted and translated to interior environments based on Lynch’s (1960) work on outdoor environments. Floor plan configuration and signs were among the four variables proposed by Weisman (1981). The two other variables proposed by Weisman (1981) are “the ability to see through or out of a setting” (p. 191) and architectural differentiation, both of which were considered as environmental attributes rather than environmental elements by the authors of the current paper. The definitions of the nine sub-domains of environmental factors are provided in Table 1.

#### Floor Plan Configuration

A floor plan configuration is the general layout of an environment (O’Neill, 1991a). Fifteen studies were found that examined the relationship between floor plan configuration and spatial memories, behavioral performance, and navigation pattern.

##### The relationship between floor plan configuration and spatial memories

Studies have examined the effects on spatial memories of several attributes of floor plan configurations: complexity, legibility, geometry, and etc. Haq and Zimring (2003) found that areas of the environments with higher axial lines connectivity appeared in the sketch map of participants more frequently. “Connectivity is a local measure that describes the relationship of each space to its immediate neighbors” (Haq and Zimring, 2003, p. 139). Accordingly, they concluded that connectivity and cognitive maps are correlated, and space syntax measures can be used as predictors of people’s ability in performing cognitive map-related tasks (such as sketch maps and pointing tasks). Studies suggest that floor plan complexity might negatively affect the accuracy of one’s cognitive map (O’Neill, 1992). For example, O’Neill (1991c) examined the effect of floor plan complexity as measured by InterConnection Density (ICD)—the total number of choices at decision points divided by the number of decision points in the layout—and found that the accuracy of acquired cognitive maps (measured using a sketch-map task) decreased as ICD increased. Slone et al. (2015) observed a similar effect, finding that participants’ sketch-maps were more accurate for environments with lower ICD values.

In addition to ICD, Li and Klippel (2016) used two space syntax measures (isovist connectivity and axial connectivity) to measure environmental legibility with the aim of determining the effect of environmental legibility on the acquisition of survey knowledge. The original authors of this paper did not provide exact definitions for isovist connectivity and axial connectivity. Readers should read the original paper to get a sense of how they defined them. Environmental legibility is defined as the degree to which patterns in an environment can be grasped and understood (Lynch, 1960). The authors obtained contradictory results, however, and concluded that their environmental measures may have failed to capture other important environmental attributes.

One class of environmental attributes that might have been missed in the aforementioned studies are geometrical attributes. Werner and Schindler (2004) argue that while different floor plans may have the same topological values (such as ICD), they may differ in their geometrical attributes. In one example, they describe a linear corridor and a spiral corridor that have the same ICD values but different levels of geometrical complexity to show that two very different geometries can indeed have the same ICD values. They examined the effect on spatial memories of a geometrical misalignment between an elevator region and other parts of a building—the elevator region was rotated 45 degrees relative to the other parts—and found that the misalignment decreased the acquisition of survey knowledge (measured via a pointing task) compared to a geometrically aligned layout. They also examined two different corner shapes—perfect orthogonal and clipped—and found that clipped corners negatively affected the acquisition of survey knowledge (Werner and Schindler, 2004). In another study, Abu-Obeid and Abu-Safieh (2010) investigated the effect on spatial memories of two different types of intersection between a corridor and a transition hall: (a) a right-angled intersection and (b) an oblique-angled intersections. They found that the participants who encountered the oblique-angled intersection performed better in a series of spatial memory tasks: a sketch-map task, an environmental recall task, and a snapshot recognition task. The authors attributed this result to an increase in visual access to the transition hall in the oblique-angled intersection. However, the authors reported that their results contradicted those of Werner and Schindler (2004), and they explained this contradiction by observing that their study had a different focus than did Werner and Schindler (2004). While Werner and Schindler (2004) examined the angles of corridors in relation to other directly connected corridors, Abu-Obeid and Abu-Safieh (2010) examined the angles between corridors and transition halls between those corridors.

Baskaya et al. (2004) compared the effects on spatial memory acquisition of symmetrical units (clinical units arranged regularly in a symmetrical layout) and asymmetrical units (clinical units arranged on one side of a main corridor in an asymmetrical layout) and found that more people reported feeling lost in the symmetrical setting, and people in the asymmetrical setting were more likely to succeed in a sketch-map task. However, because the two settings differed in many ways other than their level of symmetry, these results should be interpreted with caution. Another study investigated the effect of environmental structure on acquired spatial memories using two virtual mazes: (1) a symmetrical maze with intersections of 45 and 90 degrees and (2) an asymmetrical maze with intersections of various angles (Jansen-Osmann et al., 2007). No statistically significant results were found. Although this study was conducted in VE, it has implications in interior wayfinding.

In another study, Lu and Ye (2019) found evidence that participants acquired volumetric survey knowledge in a multilevel shopping mall where atriums connect different levels visually. Volumetric survey knowledge was defined as memorizing a building as an integrated three-dimensional whole rather than a collection of individual floors (Lu and Ye, 2019).

##### The relationship between floor plan configuration and behavioral performance

Studies suggest that floor plan complexity negatively affects wayfinding performance (O’Neill, 1991b, 1992). O’Neill (1991c) investigated the effect on wayfinding behavior of floor plan complexity (as measured by ICD) and found that as the complexity of a floor plan increased, so did wayfinding performance time, number of wrong turns, and backtracking. A subsequent analysis suggested that this relationship is mediated by cognitive-map accuracy. Slone et al. (2015) also found that completion time and error were higher in environments with higher ICD values.

In addition to ICD, Li and Klippel (2016) used two space syntax measures (i.e., isovist connectivity and axial connectivity—for definitions please refer to the actual article) to measure environmental legibility (EL) and found that participants traveled shorter paths in an environment with both high global legibility (a measure of the EL of an entire floor) and high local legibility (a measure of the EL of a specific location on a floor) than they did in an environment with high global legibility but low local legibility. Also examining data from space syntax analysis, Hölscher et al. (2012) found an association between step depth to goal (i.e., the number of turns required between an origin and a target on the shortest path between them) and the difficulty of a wayfinding task, such that routes with higher step depth to goal values were associated with lower completion speeds and longer traveled paths.

Werner and Schindler (2004) investigated the effect of geometrical misalignment on navigation time and found that participants had shorter completion times when an elevator region was aligned with other parts of a building. The same study examined the effect on behavioral performance of orthogonal and clipped corners and found no meaningful pattern. In another study, Jansen-Osmann et al. (2007) investigated the effect of environmental structure on wayfinding behavior by asking participants to navigate one of two mazes: (a) a symmetrical maze with intersections of 45 and 90 degrees and (b) an asymmetrical maze with intersections of various angles. No statistically significant results were found.

##### The relationship between floor plan configuration and navigation pattern

Haq and Zimring (2003) examined the correlation between the navigation pattern of people and measures of the environments in three hospitals. They found that people who were unfamiliar with the environments navigated on areas of the building with higher local topological properties (e.g., higher nodes recognized values) and as they became more familiar with the environments they navigated on areas with higher global topological properties (e.g., higher node integration-max values). Node recognized refers to the number of decision points that can be seen from a node; and node integration-max “is a global measure that takes into account all the spaces and hence all the steps or turns required to go from one space to all others in a spatial system” (Haq and Zimring, 2003, p. 139). In a follow-up study, Haq (2003) found that integration-3 is a better predictor compared to integration-max. “Integration-3 measures the relationship of one space to others up to three steps or turns away from it” (Haq, 2003, p. 842). Hölscher et al. (2006) found that while people who were unfamiliar with a building were more likely to use the central point heuristic, people familiar with the building were more likely to use the floor heuristic. Later, Hölscher et al. (2012) compared the traveled paths of familiar and unfamiliar people from the space syntax perspective. They found that individuals who were unfamiliar with a building were more likely to use areas of the entrance hall and its associated floor that have higher connectivity and integration values, and also they used stairs with a higher value of integration. Connectivity is a local measure of the amount of space directly visible from a node and integration refers to the degree to which a node is central in a system of nodes (Hölscher et al., 2012). This result was consistent with those of Peponis et al. (1990), who found an association between individuals’ search patterns and spaces’ degrees of integration, such that people used more integrated spaces when in doubt. Lu and Ye (2019) studied participants’ search patterns in a multilevel shopping mall where atriums connect different levels visually and they found that participants tended to use routes that were central in the building as a whole rather than the ones that were central within each floor.

#### Regions

Regions are sections of a building that has some common characteristics, and people recognize regions within a building and mentally move into them. This concept was adapted from Lynch’s (1960) definition of districts in outdoor environments. Three studies were found that examined the role of regions in relation to spatial memories and spatial updating.

##### The relationship between regions and spatial memories

In a series of experiments, Wang and Brockmole (2003) found that participants did not necessarily incorporate existing spatial memories acquired form a larger region (i.e., university campus) with the newly acquired spatial memories from a smaller region within the larger one (i.e., a room in a campus building). However, Montello and Pick (1993) found that people can integrate survey knowledge acquired from two separately learned districts together. They found that when participants were provided a verbal description of the spatial relationship between two levels of a building that they had learned separately, they could point toward landmarks on the other level (an indicator of survey knowledge), albeit with less accuracy than pointing toward landmarks on the same level.

##### The relationship between regions and spatial updating

In a series of experiments, Mou and Wang (2015) evaluated the spatial-updating accuracy of individuals using one of two strategies—piloting or path integration—while moving from one region to another. They found that the participants who used path integration updated more accurately than did the participants who used piloting.

#### Edges

Adapted from Lynch’s (1960) definition of edges in outdoor environments, in this paper edges are defined as boundaries between two regions in interior environments that are not considered paths. Only one study was found that examined the effects of edges on spatial memories.

##### The relationship between edges and spatial memories

Two major properties of edges are encoded during spatial learning: (a) global geometric cues—i.e., the overall shape of the boundary of the environment (e.g., rectangular, circular, etc.)—and (b) local geometric cues—i.e., the geometrical properties of a region within the environment (e.g., a corner of a room with a shorter wall on the left and a longer wall on the right) (Buckley et al., 2016). In a series of experiments, Buckley et al. (2016) found that participants relied on local geometric cues to find a target rather than global geometric cues. Also, they found that local geometrical cues (corners with walls of different lengths on each side) and non-geometrical cues (walls of different colors) compete to be encoded as the signal of a target, such that one who learns the location of a target based on one type of cue might be blocked from subsequent learning about the other type of cue.

#### Paths

Adapted from Lynch’s (1960) definition of paths in outdoor environments, in this paper paths are defined as channels in interior environments that people typically or potentially move along. Seven studies were found that examined relationships between paths and navigation patterns and psychological states.

##### The relationship between paths and navigation pattern

Butler et al. (1993) investigated individuals’ route preferences with respect to (1) the complexity of the route (e.g., the number of turns, the number of decision points, and the information load) and (2) the necessary energy expenditure (e.g., the distance, the number of stairs, and the availability of an elevator). Although the study did not examine participants’ actual navigation patterns, participants’ self-reported data revealed that they preferred paths that minimized energy expenditure. The paths they preferred shared three attributes: being short, having an elevator, and remaining inside the building. Hidayetoglu et al. (2012) examined the effects of color, light intensity, and light temperature on how people evaluated corridors and found that warmly colored corridors were rated more memorable, coolly colored corridors were rated more navigable, and brightly lit corridors were rated more positively.

Frankenstein et al. (2012) found that participants preferred paths with longer lines of sight, and in a similar study, Wiener et al. (2012), analyzed gaze behavior data during wayfinding and found that when participants were asked to choose a path toward a goal in a VE, they were most likely to choose the path that had the longest line of sight. Different attributes of corridors and choice pattern of people at intersections were examined in two studies and findings showed that in the absents of signs, people are more likely to choose wider and brighter corridors at T-type intersection (nodes with two alternatives of turning left or right), but not necessarily at F-type intersections (nodes with two alternatives of continuing straight or turning right/left) during non-emergency situations. However, people relied on signs when they were available (Vilar et al., 2014b, 2015).

##### The relationship between paths and psychological state

In an emergency and stressful condition, the results of two studies suggested that people tended to follow brighter corridors in both T-type and F-type intersections and wider corridors are more preferred in only T-type intersections (brightness was found to be a stronger factor than width) when signs were not available (Vilar et al., 2013, 2014b). In a follow-up study, Vilar et al. (2014b) found that during emergency egress, people did not necessarily follow exit signs.

#### Nodes

Nodes are decision points at which two or more alternatives are available (O’Neill, 1991a). Six studies investigated navigation pattern and psychological state in relation to nodes.

##### The relationship between nodes and navigation pattern

In a series of experiments, Van Tilburg and Igou (2014) examined which of four heuristics—the action-continuation heuristic, the initial segment heuristic, the least-angle heuristic, and the hill-climbing heuristic—individuals preferred while navigating a maze with equally functional routes at a choice point and found that participants behaved in a way consistent with use of the action-continuation heuristic. In three other studies, navigation patterns of people were investigated in two types of intersections: T-type intersections (nodes with two alternatives of turning left or right) and F-type intersections (nodes with two alternatives of continuing straight or turning right/left) (Vilar et al., 2012, 2014b, 2015). Findings showed that the type of intersection affected people’s behavior, such that brighter corridors were preferred at both T-type and F-type intersections and wider corridors at only T-type intersections.

##### The relationship between nodes and psychological state

Tang et al. (2009) conducted a study in Taiwan (where people drive on the right side of the road) and found that during emergency egress, participants tended to turn left at a T-type intersection (i.e., a node with two alternatives, left and right). Two other studies investigated the navigation pattern of people in emergency and stressful situations at T-type and F-type intersections and they also found that intersection type affected the behavior of people (Vilar et al., 2013, 2014b).

### Landmarks

Landmarks are objects, places, and environmental attributes that serve as reference points (Lynch, 1960). Landmarks are remembered due to their visual appearance, subjective importance (Iachini et al., 2009), or strategic function (Lynch, 1960). Fourteen studies examined the relationships between landmarks and spatial memories, spatial updating, logical associations, neuroanatomy, behavioral performance, age, and sex.

#### The relationship between landmarks and spatial memories

Hidayetoglu et al. (2012) investigated the effects of different attributes of corridors— color, light intensity, and light temperature—and found that warm colors were rated as more memorable. For this reason, warm colors might be used as landmarks (Hidayetoglu et al., 2012). Davis et al. (2008) examined the effects of visual attributes of landmarks on women’s place-learning performance (measured via initial heading direction and completion time) in three conditions: simple salient (which featured four simple drawings of black objects against a white background mounted on black walls), complex salient (which featured walls of different materials, with different textures, and with realistic photographs of objects), and non-salient (which featured white walls decorated with a gray moon landscape and an abstract picture). Place learning is “the cognitive process involved in encoding the cognitive map” (Davis et al., 2008, p. 253). The authors found that while the participants in the “simple salient” condition initially exhibited better place learning, over time, the participants in the “complex salient” condition performed better. The participants in the “non-salient” condition performed the worst. These results suggest that landmarks with visual details may facilitate place learning (Davis et al., 2008). In a related study, Werkhoven et al. (2014) found that a combination of auditory and visual landmarks improved the acquisition of spatial memories to a greater degree than did either auditory or visual landmarks alone.

Jansen-Osmann and Wiedenbauer (2004) examined the effect of color coding on the survey-knowledge acquisition of individuals navigating a virtual maze in one of two conditions: (1) a condition in which different regions had different ground colors and (2) a condition in which different regions had the same gray ground color. They found that although the participants in the “color” condition exhibited better wayfinding performance (as measured by traveled distance) than did the participants in the “same-color” condition, the participants in both conditions acquired the same degree of survey knowledge. Although this study was conducted in VE, it has implications in interior wayfinding. In another study, Jansen-Osmann and Fuchs (2006) examined the effect of landmarks on spatial-knowledge acquisition (measured via a detour task, direction estimation, and a map task) and found no effect.

Sameer and Bhushan (2015) evaluated the effectiveness of a modified version of the loci method (i.e., a memorization technique to remember unfamiliar routes by using familiar concepts) in helping participants to remember a route. Participants were assigned to one of two conditions: (1) a “no-landmark” condition and (2) an “imaginary landmarks” condition (which implemented the loci method). In the imaginary landmark condition, participants were asked to generate a list of familiar items to be used as imaginary landmarks and to associate each item with an action at a decision point (e.g., going left, right, or straight). The results revealed that the participants in the no-landmark condition exhibited better route memory.

#### The relationship between landmarks and spatial updating

Wan et al. (2012) found that when several landmarks can serve as potential return targets, keeping track of all of them can increase one’s processing load. Specifically, they found that participants who were not informed to which landmark they should have returned before beginning a wayfinding task, identifying the location of return targets took longer compared to the informed group. However, no difference was observed in other measurements, including direction error, distance error, and position error.

#### The relationship between landmarks and logical associations

Logical associations refer to people’s inferences regarding typical “links between object types and certain functions or regions in buildings” (Frankenstein et al., 2012, p. 165). Frankenstein et al. (2012) found that background knowledge can affect wayfinding decisions. They found that novice participants used their prior knowledge to infer possible logical associations between observed landmarks and targets in a building. For example, participants’ ratings suggested that a stand-up display and an auditorium, an elevator and a main entrance, and a metal door and a cellar entrance were more likely to be seen together.

#### The relationship between landmarks and neuroanatomy

Sharma et al. (2017) analyzed EEG data and found that an environment with lots of landmarks activated the left hemisphere (specifically the left posterior inferior and superior regions) to a greater degree than did an environment without landmarks. In another EEG study, Kober and Neuper (2011) found that women demonstrated more theta oscillations than did men during landmark processing.

A study by Janzen and Jansen (2010) suggests that similar landmarks (such as fire extinguishers and mailboxes) can cause confusion when they appear multiple times during wayfinding; people can become confused regarding which landmark is the signal for which action at which decision point. Janzen and Jansen (2010) also analyzed fMRI results and found that a neural mechanism exists that distinguishes between useful and misleading landmarks. They found that useful landmarks are represented in the parahippocampal gyrus, while the right middle frontal gyrus is responsible for resolving confusion over which landmark signals which action.

#### The relationship between landmarks and behavioral performance

Sharma et al. (2017) found that participants made fewer errors and took less time when they navigated an environment with multiple landmarks than when they navigated an environment without landmarks. In another study, Werkhoven et al. (2014) found that participants demonstrated better wayfinding performance (as measured by traveled distance and travel time) when auditory and visual landmarks were combined than when either auditory or visual landmarks were present in isolation. Jansen-Osmann and Wiedenbauer (2004) examined the effects of color coding on the wayfinding performance of participants navigating a virtual maze in one of two conditions: (1) a condition in which different regions had different ground colors and (2) a condition in which different regions had the same gray ground color. They found that although traveled distance decreased across learning trials, it decreased faster in the “color” condition than in the “same-color” condition. Although this study was conducted in VE, it has implications in interior wayfinding.

#### The relationship between landmarks and age

Davis et al. (2008) examined the effects of the visual attributes of landmarks on the place-learning performance (as measured by initial heading direction and completion time) of older and younger women across three conditions: (1) a “non-salient” condition featuring white walls decorated with a gray moon landscape and an abstract picture, (2) a “simple salient” condition featuring four simple drawings of black objects against a white background mounted on black walls) (3) and a “complex salient” condition featuring walls with different materials and textures and realistic photographs of objects. The results showed that younger women outperformed older women in place learning task. Participants performed best in the complex salient condition (which was rich in color, texture, and detail) and worst in the non-salient condition (which had the fewest details). The older women’s difficulty in the non-salient condition relative to their younger counterparts was so dramatic which suggests that older women relied on salient landmarks more that younger women.

#### The relationship between landmarks and sex

In an EEG study, Kober and Neuper (2011) found that women demonstrated more theta oscillations than did men during landmark processing. In addition, Choi et al. (2006) found that women acquire spatial memories more effectively when they use landmark-biased strategies. In two studies by Schmitz (1997, 1999), women referred to landmarks more often than did men in map-drawing and direction-giving tasks. While women preferred landmarks to route directions (e.g., turn left or right), men used both landmarks and route directions.

#### Signs

Signs are elements that provide directional information in an environment (O’Neill, 1991a). Twelve studies were found that examined the relationships between signs and information pick-up, behavioral performance, navigation pattern, psychological state, and culture.

##### The relationship between sign and information pick-up

Rousek and Hallbeck (2011) investigated the comprehensibility of signs in healthcare facilities and found that the following improved the comprehensibility of such signs: (a) the consistent use of pictograms throughout the environment to represent concepts and actions, (b) the inclusion of human figures that are neither too abstract nor too complex, and (c) signs with greater color contrast. In a study by Hashim et al. (2014), participants from the United Arab Emirates experienced greater difficulty understanding healthcare signs (e.g., signs for outpatient clinics and gynecology) than they did understanding general-purpose signs (e.g., signs for stairs and restrooms). Level of education, age, and culture were also found to be related to participants’ understanding of healthcare signs.

##### The relationship between sign and behavioral performance

Butler et al. (1993) examined the effects of signage and you-are-here maps (YAH maps) on wayfinding behavior and found that signage was a more effective system of wayfinding aid, such that participants found a target destination faster in the signage condition than in the YAH map condition. Other studies support this conclusion (e.g., Wright et al., 1993; Chen et al., 2009). Wright et al. (1993) found that participants who used signage in a hospital were faster than participants who used a hand-held map in addition to signage, although the participants in the signage-only condition demonstrated more retracing behaviors to make sure that they were on the right track. In another study, O’Neill (1991b) found that signage did not compensate for wayfinding issues caused by increased floor-plan complexity.

O’Neill (1991b) investigated the effects on wayfinding performance of different types of signage using three conditions: a condition featuring graphic signs, a condition featuring textual signs, and a condition with no signage. The results revealed that the participants in the graphic-sign condition navigated the fastest, while the participants in the no-signage condition navigated the slowest. The participants in the textual-sign condition exhibited the least backtracking and made the fewest wrong turns, followed by the participants in the graphic-sign condition and the participants in the no-signage condition. In another study, Cope et al. (1999) found that signs that included both words and icons produced the shortest completion times and traveled distances, followed by word-only signs and icon-only signs. Vilar et al. (2014a) investigated how wayfinding behaviors differed when two different types of signage systems were used: (a) color trails on the floor and a wall-mounted, color-coded signage panel and (b) a wall-mounted signage panel with directional arrows. They found that signage improved wayfinding performance and that the signage system featuring the colored trails produced fewer pauses, shorter traveled distances, shorter completion times, and higher speeds, although the difference was not statistically significant.

##### The relationship between sign and navigation pattern

In two studies, it was found that people prefer to follow wider and brighter paths when several alternatives were available. However, when information provided by signs and people’s preference conflicted, people were more likely to follow signs (Vilar et al., 2014b, 2015).

##### The relationship between sign and psychological state

Evidence suggests that signs reduce the required time for emergency egress. Tang et al. (2009) found that when confronted with a door and an emergency exit sign pointing away from the door, half of the participants used the door instead of following the exit sign, even though the door was not marked as an exit. Several studies reported that in non-emergency situations, people preferred to follow brighter and wider corridors at intersections when signs were not available, although, they followed signs if available (Vilar et al., 2014b, 2015). However, Vilar et al. (2014b) found that in emergency and stressful situations, participants did not necessarily follow signs, and some relied on environmental affordances (e.g., brighter and wider corridors).

##### The relationship between sign and culture

Joy Lo et al. (2016) examined the comprehensibility of a universal wayfinding healthcare signage system developed in the United States (Hablamos Juntos) in Taiwan and found that participants had difficulty understanding its symbols. Similar results were obtained by another study involving participants from the United Arab Emirates (Hashim et al., 2014). In both studies, level of education and culture were noted as contributing factors to participants’ understanding of healthcare signs (Hashim et al., 2014; Joy Lo et al., 2016).

#### Maps

Maps are “diagrammatic, 2-dimensional representation of the global environment” (Pati et al., 2015, p. 50). Ten studies were found that examined the relationship between maps and spatial memories, information pick-up, and behavioral performance.

##### The relationship between maps and spatial memories

Richardson et al. (1999) compared the acquired spatial knowledge of participants in three learning conditions: map, real-world navigation, and virtual environment. Although they did not find statistically significant differences between the acquired spatial knowledge (measured via a pointing task) of the participants in each condition, they found that the participants in the virtual-environment condition acquired less accurate spatial knowledge. They also found that the performance of the participants in the map condition declined when the orientation of the map in the learning phase and the participants’ testing orientation were not aligned. In an older study, Thorndyke and Hayes-Roth (1982) found that individuals who used maps performed better than real-world navigators in estimating the relative locations of and straight-line distances between landmarks, but not in estimating route distances and pointing to unseen objects. This difference disappeared as exposure to the environment increased (Thorndyke and Hayes-Roth, 1982). Golledge et al. (1995) obtained similar results, finding that participants who learned from a map tended to outperform participants who learned by navigating a virtual environment (VE) in spatial knowledge tasks (e.g., orientation and distance estimation), although this difference was not statistically significant.

##### The relationship between maps and information pick-up

Hölscher et al. (2009) found that novice wayfinders used wall-mounted maps more often than did individuals who were familiar with a building but that use of maps did not fill the spatial knowledge gap between the two groups. Meilinger et al. (2006) examined the impact on wayfinding of providing different levels of spatial knowledge using two conditions: (1) a condition in which participants were provided a standard floor plan that offered survey knowledge and (2) a condition in which participants were provided a highly schematic map that conveyed only route knowledge. They found that because the participants in the floor-plan condition spent longer reading their maps, they took longer to reach the target location.

##### The relationship between maps and behavioral performance

Several studies found that using maps was time-consuming and negatively impacted wayfinding performance (e.g., Butler et al., 1993; Wright et al., 1993; Hölscher et al., 2009). Butler et al. (1993) compared the effects of signage and you-are-here maps (YAH maps) on wayfinding performance and found that the participants in the YAH-maps condition took longer to find a target. Wright et al. (1993) found that participants who used signage in a hospital were faster than participants who used signage and a hand-held map, although the participants in the signage-only condition demonstrated more retracing behaviors to make sure they were on the right track. Chen et al. (2009) also found that signs were more effective than YAH maps, but they also found that availability of signage for female participants could eliminate sex differences in wayfinding performance (as measured by navigation time). Münzer and Stahl (2011) examined the impact on wayfinding performance of three learning conditions: (1) a route was illustrated on a map, (2) a series of images of decision points, and (3) a virtual walk of the route. They found no difference between the behavioral performance of participants in map condition and the other two conditions together. However, participants performed better in the virtual walk condition compared to the condition in which they were exposed to a series of images of decision points.

Levine et al. (1984) found that YAH maps that were misaligned with a building made it more difficult for participants to solve a wayfinding problem and increased the time it took them to study the maps. Meilinger et al. (2006) examined the impact on wayfinding performance of providing different levels of spatial knowledge using two conditions: (1) a condition in which participants were provided a standard floor plan that offered survey knowledge and (2) a condition in which participants were provided a highly schematic map that conveyed only route knowledge. They found that the participants who used the schematic map to find a target in a building exhibited better wayfinding performance (i.e., shorter completion times and shorter route distances). However, the participants who used the standard floor plan performed better when asked to navigate a smaller part of the building, and the results were not statistically significant. In addition, the participants in both groups performed equally well in a self-localization task (a task in which they were disoriented and asked to identify their location relative to their surrounding environment) (Meilinger et al., 2006).

#### Other Environmental Factors

Other environmental factors include “elements or attributes of physical environment procured or designed by interior designers used in a different way by subjects” (Pati et al., 2015, p. 50). Three studies examined other environmental factors in relation to logical associations and information pick-up.

##### The relationship between other environmental factors and logical associations

Frankenstein et al. (2012) found that individuals use general background knowledge to compensate for deficiencies in spatial knowledge. In their study, they asked participants to rate multiple locations within a public building (an auditorium, restrooms, a broom closet, etc.) on three dimensions: central/peripheral area, public/non-public area, and busy/not busy area. They found that the participants assumed the following logical associations: (1) public areas were in central locations and non-public areas were in peripheral locations; (2) auditoriums, main exits, and restrooms were in central and busy locations; (3) broom closets, main exits, and entrances to cellars were in not-busy and peripheral locations; (4) some landmarks (such as chairs and waiting areas) were in public locations; (5) some landmarks were adjacent to other locations due to their functions (e.g., elevators were next to main entrances).

Pati et al. (2015) examined various elements of the environment that contribute to wayfinding in healthcare environments. Of the environmental elements found to support wayfinding, two related to logical associations about the building: (a) functional clusters and (b) interior element pairing. “Functional clusters are defined as logical clustering of programmatic spaces with (sometimes) mutually supportive or complementary functions” (Pati et al., 2015, p. 51), for example, main entrances and admissions, cafeterias and lobbies, and waiting areas and children’s play areas. “Interior element pairing” was defined as the “logical pairing of interior architecture elements (excluding furniture), with associated functions” (Pati et al., 2015, p. 52). For example, participants logically considered the concurrent presence of counters and interior windows as an indicator of admissions. Type of furniture was found to be another environmental factor that participants relied on to make logical conclusions. For instance, seats were considered an indicator of a waiting area (Pati et al., 2015).

##### The relationship between other environmental factors and information pick-up

Pati et al. (2015) identified environmental elements used as sources of information during wayfinding in a healthcare setting. They found that participants used the following elements as sources of information (ordered from highest to lowest based on frequency of use): signs, architectural features (i.e., view to outside and multilevel interior view from atrium), maps, interior elements (e.g., artwork, display boards, and information counters), logical clusters of functions, pairings of interior elements, structural elements, and furniture. In another study, Ghamari and Pati (2018) analyzed eye-fixation data obtained during wayfinding and found that signs, architectural features, maps, and interior artifacts most frequently attracted the attention of participants, in conformity with Pati et al. (2015) study findings.

## Discussion

A review of 84 studies showed that wayfinding is a complex phenomenon that involves multiple cognitive processes and behaviors. Two broad factors were identified in the reviewed studies which are (a) user factors and (b) environmental factors. User factors were classified into three domains which are (1) wayfinding cognition, (2) wayfinding behavior, (3) individual and group differences. Environmental factors have two domains which are (1) environmental elements and (2) environmental cues. Many of these domains include several sub-domains, and most of the studies focused on multiple sub-domains.

Spatial cognition is a much broader issue than wayfinding. There are some areas of research that only lead to general spatial cognition knowledge and not wayfinding knowledge. However, some areas of spatial cognition research do have implications in wayfinding. Of the cognitive functions related to wayfinding, spatial memories have received the most attention in published literature. Gaining spatial memories can improve people’s wayfinding performance, but providing different levels of spatial memories (e.g., route knowledge versus survey knowledge) can have different effects on behavioral performance. While both verbal and spatial working memories are involved in the acquisition of spatial knowledge, spatial working memory seems to play a more critical role. Studies in the neuroscience literature suggest that different parts of the brain are responsible for handling different types of spatial knowledge. In addition, there seems to be an association between the acquisition of survey knowledge and the use of path integration. Anxiety resulting from being in a new environment (neophobia) seems to affect people’s navigation patterns. For example, in one study, people tended to stay closer to the borders of an arena in the early and middle phases of learning a new place. Aging can negatively affect the acquisition of spatial memories, and sex differences are apparent in the acquisition of spatial memories. Men tend to outperform women in tasks that require survey knowledge, while women tend to rely on landmark-biased strategies.

Regarding spatial reference frames, one study found no difference in the behavioral performance of people who learned a new environment through either allocentric or egocentric visualizations. Older adults seem to rely on the egocentric reference frame to a greater degree than do younger adults, and evidence suggests that while women prefer the egocentric reference frame, men prefer the allocentric reference frame.

Regarding spatial updating, a correlation was found between the acquisition of survey knowledge and path-integration. Also, evidence suggests that when moving from one region to another, path integration can yield more accurate spatial updating than can piloting.

Regarding information pick-up, receiving different levels of knowledge can produce different levels of behavioral performance. In one study, those who used route descriptions (e.g., turns and landmarks) performed better than did those who used survey descriptors (e.g., cardinal directions and distances). Aging was also found to affect the type of information that people look for, and older adults seem to prefer more salient environmental information. Sex differences were also evident in information pick-up. While women preferred to pick up landmark cues, men preferred to pick up geometry cues.

Regarding spatial abilities, a neuroscience study found that people who considered themselves good navigators differed in their neuroanatomy from people who considered themselves bad navigators. In addition, people who reported having a good sense of direction tended to be faster wayfinders and to commit fewer errors. Aging negatively affects spatial abilities, thereby reducing wayfinding performance.

Data on sex differences in wayfinding behavioral performance are inconsistent. While some studies found no differences, other studies showed that men outperformed women in, for example, speed and taking the shortest route.

In studies focused on individual and group differences, age, sex, culture, and psychological state were found to influence different aspects of wayfinding. Evidence suggests that women experience more spatial anxiety than do men and that while stress does not affect women’s landmark-guided navigation, it does disrupt their cognitive-map-guided navigation.

In the domain of physical environment, the literature identified several environmental factors that can influence wayfinding (i.e., floor plans configuration, regions, edges, paths, nodes, and landmarks, signs, maps, etc.). In relation to the floor plan configuration, the complexity of a floor plan (as measured by its topological attributes) can negatively affect the accuracy of individuals’ cognitive maps. Evidence also suggests that geometrical attributes (such as angles between different parts of a building, symmetrical/asymmetrical layouts, and angles between corridors and intersection halls) can influence the acquisition of survey knowledge. People seem to be able to memorize multi-level buildings with an atrium as an integrated three-dimensional whole rather than a collection of individual floors. Also, people tend to use routes that are central in the building as a whole rather than the ones that are central within each floor. Floor-plan measures such as ICD, isovist connectivity, axial connectivity, and step depth to goal were also found to be associated with wayfinding behavioral performance. In addition, measures such as connectivity and value of integration can predict people’s navigation patterns.

Studies regarding regions are scarce. Findings regarding the ability of people to integrate spatial knowledge acquired separately from different regions have some discrepancies. More importantly, path integration was found to be a more effective spatial updating strategy than piloting when moving from one region to another.

In case of edges, the current review found only one study into edges, and this study found that local geometric properties of edges can be used as cues in spatial learning and that non-geometrical cues (such as wall color) can compete with local geometric cues to be encoded as the signal for a target.

In relation to paths, studies found that people prefer paths that (a) minimize energy expenditure, (b) have the longest line of sight, and (c) are wider and brighter than available alternatives. In addition, corridors with warm colors were viewed as more memorable, corridors with cool colors were rated more navigable, and brighter corridors were perceived more positively.

In the case of nodes, the type of intersection (T-type vs. F-type) seems to influence people’s path preference, such that brighter corridors were preferred at both T-type and F-type intersections and wide corridors were preferred at only T-type intersections. In one study, participants in an emergency egress situation tended to turn left at a T-type intersection (this study was conducted in Taiwan where people drive on the right side of the road). In addition, people tend to use the action-continuation heuristic when faced with equally functional routes.

Landmarks that provided more visual detail and combined auditory and visual information were found to improve some aspects of spatial memories. However, keeping track of multiple landmarks as possible return targets imposed a higher processing load during spatial updating. In one study, prior knowledge was found to be important in inferring possible associations between observed landmarks and a target in a building. One neuroscience study suggests that a neural mechanism exists that distinguishes useful and misleading landmarks. Overall, the presence of landmarks improved wayfinding performance.

In the case of signs, one study showed that signage did not compensate for wayfinding issues caused by an increase in floor-plan complexity. However, signage was found to be more effective than YAH maps. Three factors were identified that can improve the comprehensibility of signs in healthcare facilities: (a) the consistent use of pictograms throughout the environment to represent concepts and actions, (b) the inclusion of human figures that are neither too abstract nor too complex, and (c) signs with greater color contrast. Level of education, age, and culture were identified as factors that contribute to participants’ understanding of healthcare signs. Signs that include both words and icons were found to produce the shortest completion times and traveled distances, followed by signs that include only words and signs that include only icons. Evidence also suggests that signs reduce the time required for emergency egression. However, in emergency egression, people did not necessarily follow signs, and some relied on environmental affordances (e.g., brighter and wider corridors).

Studies into maps showed that learning an environment from a map is more effective than learning by navigating a virtual environment. However, several studies found that using maps was time consuming and negatively impacted wayfinding performance. In addition, using a misaligned map was found to make it more difficult to solve a wayfinding problem, and using a schematic map was found to yield better performance than using a highly detailed one. One study found no difference between the behavioral performance of participants who learned an environment through a map on which a route was indicated compared to who learned through a virtual walk or a series of images of that route.

Few studies investigated environmental factors used in wayfinding. Among the environmental factors identified as contributing to wayfinding were signs, architectural features (i.e., view to outside and the multilevel interior view from the atrium), maps, interior elements (e.g., artwork, display boards, and information counters), logical clusters of functions, pairings of interior elements, structural elements, and furniture. Background knowledge was found to play an important role when people’s spatial knowledge is insufficient, such that people infer logical associations based on their prior knowledge.

Taken together, the findings of the studies presented here provide evidence that the environment plays an important role in both the cognitive and behavioral aspects of wayfinding. Our review reveals that most of the literature on environmental factors focuses on landmarks, signage, floor plan configuration, and maps. Environmental factors, such as regions, edges, and nodes, have not been studied as extensively. While the relationships between environmental factors and spatial memories and behavioral performance are a major focus of the literature, the effects of environmental factors on other cognitive functions, such as spatial reference frames and spatial updating, have been neglected. In addition to studies examining causal relationships, more exploratory studies are needed to identify other environmental factors and attributes that might impact wayfinding. Additional studies are also needed into people’s background knowledge and logical associations regarding built environments.

Although wayfinding has attracted the attention of decision makers, no single source had reviewed the different aspects of wayfinding. This review is meant to deepen the field’s understanding of the factors that contribute to wayfinding and to serve as a resource for decision makers and designers. The categorization provided in this paper is an attempt to establish a common language and to provide a framework for organizing the literature, and also suggest directions for future research.

This review suffers from a number of limitations. First, because only four databases were searched, it is possible that other relevant studies were not included. Second, conference papers were not included in this review, thus it is possible that other relevant studies were missed. Third, the search was limited by the keywords used. In the literature, wayfinding and other terms such as navigation were used interchangeably (e.g., McNamara et al., 2008). Including other keywords may yield other relevant articles. Fourth, as is the case with any literature review, studies published after our initial search were not included, so the most recent studies are not reported here.

## Author Contributions

DP conceived the study, supervised the manuscript, and revised the last version of this article. SJ and ME reviewed the literature. SJ wrote the original draft of the manuscript. All the authors contributed to the article and approved the submitted version.

## Conflict of Interest

The authors declare that the research was conducted in the absence of any commercial or financial relationships that could be construed as a potential conflict of interest.
